# Per|Mut: Spatially Resolved Hydration Entropies from Atomistic Simulations

**DOI:** 10.1021/acs.jctc.0c00961

**Published:** 2021-03-12

**Authors:** Leonard P. Heinz, Helmut Grubmüller

**Affiliations:** Department of Theoretical and Computational Biophysics, Max-Planck Institute for Biophysical Chemistry, 37077 Göttingen, Germany

## Abstract

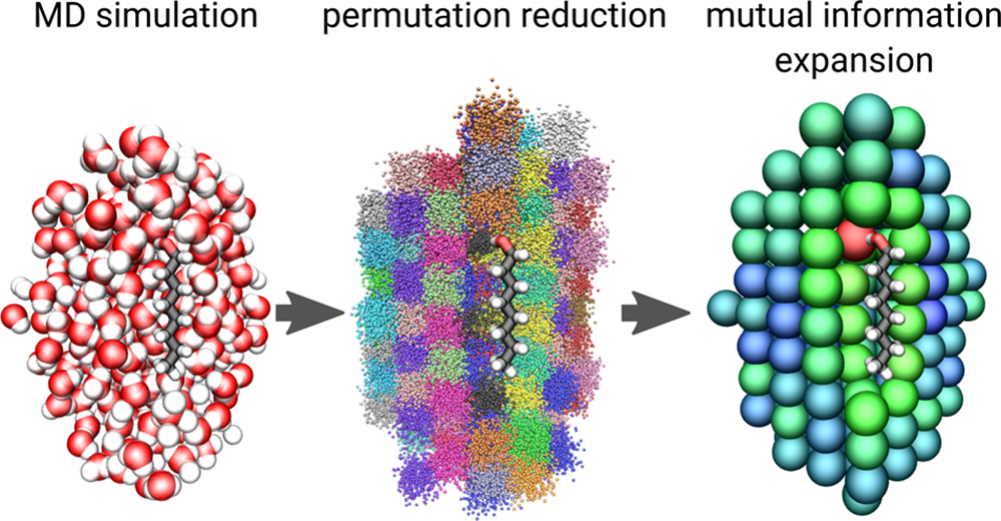

The hydrophobic effect is essential for many biophysical phenomena and processes. It is governed by a fine-tuned balance between enthalpy and entropy contributions from the hydration shell. Whereas enthalpies can in principle be calculated from an atomistic simulation trajectory, calculating solvation entropies by sampling the extremely large configuration space is challenging and often impossible. Furthermore, to qualitatively understand how the balance is affected by individual side chains, chemical groups, or the protein topology, a local description of the hydration entropy is required. In this study, we present and assess the new method “Per|Mut”, which uses a permutation reduction to alleviate the sampling problem by a factor of *N*! and employs a mutual information expansion to the third order to obtain spatially resolved hydration entropies. We tested the method on an argon system, a series of solvated *n*-alkanes, and solvated octanol.

## 1 Introduction

The thermodynamics of the hydration shell plays an important role in many biophyiscal processes, such as phase separation, membrane and micelle formation,^1−3^ or the function and folding of proteins.^4,5^ These processes are driven by the hydrophobic effect,^6−10^ which results from a delicate balance between entropic and enthalpic contributions of the first few solvent layers but is quantitatively not yet fully understood.^11^ A better understanding of the behavior of water molecules at heterogeneous surfaces is therefore necessary.

Atomistic molecular dynamics (MD) simulations have proven to reproduce the effects of hydrophobic interaction quantitatively.^2,12^ However, the lack of a straightforward way to quantify the hydration entropy contributions of specific side chains or functional groups of atoms precludes a deeper understanding of the molecular driving forces and the energetics of solvation. Further, the shallow energy landscape that governs the dynamics of the solvent molecules requires sampling of an extremely large configuration space and thus poses a severe challenge for entropy calculation.

The solvation entropy of a simple system can be calculated using thermodynamic integration (TI),^13,14^ which, however, lacks spatial resolution, and a vast amount of sampling is needed for more complex systems.

3D-two-phase-thermodynamics (3D-2PT)^15−17^ is a voxel-based approach and thus yields spatially resolved hydration entropies but relies on the assumption that the system can be treated as a superposition of gas-like and solid-like components.

Likewise, the grid cell theory (GCT)^18^ also yields solvent entropies but relies on a generalized Pauling’s residual ice entropy model^19,20^ for the rotational entropy term.

In the grid inhomogeneous solvation theory (GIST),^21−27^ the entropy is approximated by a truncated expansion of single-body and multibody correlation functions, which are calculated on a three-dimensional grid. Although the method also provides spatial resolution, the GIST expansion is usually truncated at the single-body and rarely at the two-body correlation term and therefore does not capture most multibody effects, which, as will be shown below, are important.

To address these issues, we developed an MD-based method to calculate the spatially resolved hydration entropy from atomistic simulations via permutation reduction and a mutual information expansion (“Per|Mut”), which calculates entropies directly by sampling the configuration space probability density ϱ as *S* = −*k*_B_⟨log ϱ⟩.

As described in Section 2.2, adequate sampling of the full configuration space is typically impossible, because in such an approach, the solvent particles are inherently treated as indistinguishable. The space that needs to be sampled is, therefore, *N*!-times larger than fundamentally necessary, which results in slow convergence. Whereas other methods do not suffer from this problem, as they compute entropies indirectly through derived quantities (3D-2PT) or by switching to a grid-based representation that is agnostic of the particle identities (GCT and GIST), we here address this issue by taking advantage of the permutation symmetry of the *N* identical solvent molecules to enhance configuration space sampling by the Gibbs factor *N*!. To calculate the entropy from the permutationally reduced trajectory, we use a mutual information expansion. The latter step is similar in spirit to GIST but here is based on particle positions rather than voxels, and all correlations up to three-body correlations are routinely included.

The method yields spatially resolved entropy contributions from translational and rotational degrees of freedom as well as from their correlation on a per-molecule level. The distinction between first-order entropy and contributions from many-body correlations furthermore provides an interpretation of the physical origins of solvent-driven free energy changes.

We have addressed the rotational part of solvent entropies in a previous publication^28^ and will therefore focus the theory below on the translational part and the mutual information term that describes the correlation between translational and rotational degrees of freedom. Subsequently, we will assess the accuracy and convergence on 1728 argon atoms in Section 4.1. In Sections 4.2 and 4.3, we will apply Per|Mut to hydrated systems, namely to solvated alkanes and to octanol.

## 2 Theory

### 2.1 Absolute Entropy

We separate the total entropy *S*_tot_ into the sum of contributions from the translational degrees of freedom *S*_trans_, the rotational degrees of freedom *S*_rot_, and a mutual information (MI) term −*I*_trans–rot_, which quantifies the correlation between translational and rotational motions. Note that rotational entropy is also defined as a conditional entropy by some authors,^25,27,29^ in which case, it includes the MI term −*I*_trans–rot_.

The translational entropy is
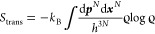
1with the Boltzmann constant *k*_B_, Planck’s constant *h*, momenta {***p***_*i*_}, and positions {***x***_*i*_} for *N* solvent molecules, the normalized and dimensionless phase space density  with the Hamiltonian , and the partiton function *Z*. The translational entropy, in turn, separates into a kinetic part, which can be calculated analytically, and the configurational contribution is
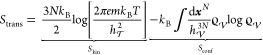
2where *m* is the mass of a water molecule,  with arbitrary , and  is the probability density in 3*N*-dimensional configuration space. Here, we present a method to calculate the total solvent entropy *S*_kin_ + *S*_conf_. Since *S*_kin_ is available analytically, we will here mainly focus on *S*_conf_.

We calculate *S*_conf_ by first carrying out an atomistic molecular dynamics (MD) simulation of solvent and solute. We then employ a permutation reduction of the identical solvent molecules as described in Section 2.2 to increase sampling by a factor of *N*!. Finally, we calculate entropies from the permuted trajectory via a third-order mutual information expansion (MIE) with a *k*-nearest-neighbor probability density estimator, as described in Section 2.3.

A MIE is also used in the same manner to estimate the correlation term −*I*_trans–rot_.

### 2.2 Permutation Reduction

The sampling of *S*_conf_, and thus the direct calculation of solvent entropy from computer simulations, generally suffers from slow convergence. Contrary to the collective motion of macromolecules, which is usually highly coupled, the diffusive motion of solvent molecules leaves almost the entire configuration space accessible, thus rendering sufficient sampling practically impossible.

The volume of the full 3*N*-dimensional translational configuration space of *N* water molecules with a constant density scales as *N*^*N*^, which renders it impossible to numerically sample the configuration space even for a small system of ∼100 water molecules. This problem arises because the concept of phase space (or configuration space) inherently assigns unique labels to physically identical water molecules. Therefore, each microstate is counted redundantly *N*! times, where the physically identical microstates only differ by a permutation of the indistinguishable water molecules, leading to a configuration space volume *N*!-times larger than physically necessary.

In an analytical treatment, this problem is, of course, solved by the Gibbs factor *N*!.^30^ For a numerical approach, however, the Gibbs factor cannot be straightforwardly applied. Permutation reduction^31,32^ solves this problem by relabeling (i.e., permuting) the solvent molecules in each frame of an atomistic trajectory such that the trajectory is mapped into a configurational subspace with a volume reduced by the Gibbs factor *N*!. Here, we summarize the aspects of permutation reduction that are relevant for the entropy estimate; an in-depth derivation is provided elsewhere.^31,32^

The alleviation of the sampling problem is achieved by choosing a permutation π for each frame of the trajectory {***x***_*i*_(*t*)} that minimizes the distance
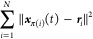
3with respect to an arbitrary but fixed set of reference positions {***r***_*i*_}.

**Figure 1:**
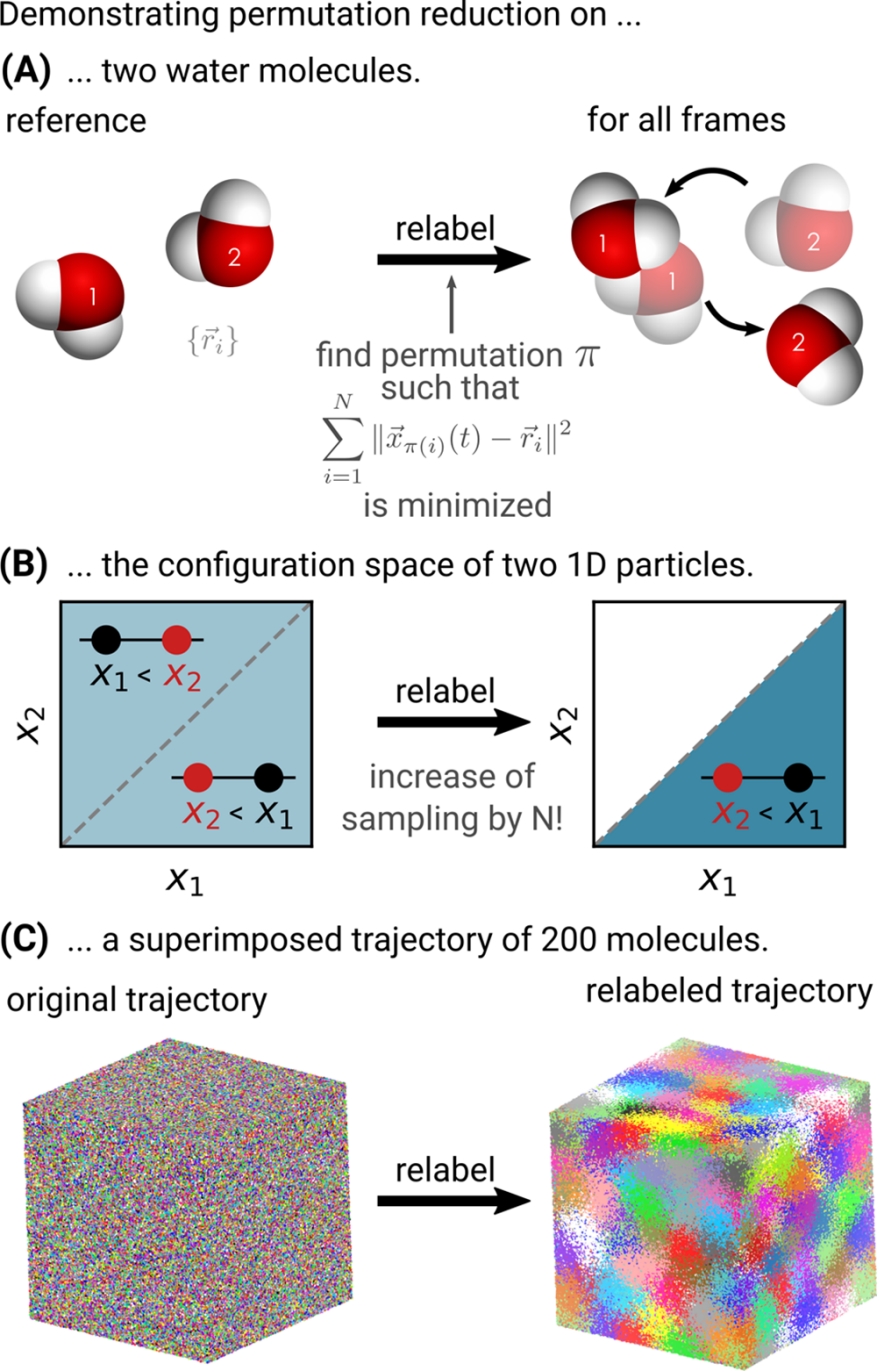
Concept of permutation reduction on (A) two water molecules, (B) two one-dimensional molecules, and (C) a superimposed trajectory of 200 molecules. In the last case, each color represents a single molecule.

Figure 1A demonstrates this approach for the simplest case of two water molecules. In the right panel, the water molecules have moved away from their reference positions (shown on the left), such that the deviation from the reference is minimized if the labels are swapped. Figure 1B visualizes the effect on the accessible configuration space for two one-dimensional molecules. Before permutation reduction, the system is either in a regime of *x*_1_ < *x*_2_ or *x*_1_ > *x*_2_, with the accessible configuration space marked in blue. After permutation reduction, the molecules are relabeled such that, depending on the reference configuration, the system remains in one of the two regimes. Thereby, the configuration space volume is reduced by a factor of 2!. Although this factor seems small, note that it is *N*! for *N* molecules and hence represents an enormous alleviation of the sampling problem for larger *N*.

Figure 1C shows the effect of permutation reduction on 3D space for a box of 200 molecules. Now, each molecule is localized to a small region centered at the reference position. Importantly, the physics of the system is unchanged.

### 2.3 Entropy Estimation

Even after permutation reduction, *S*_conf_ cannot be computed directly, because it still requires sampling of a 3*N*-dimensional space. Instead, we use a mutual information expansion^33−36^ and calculate its terms with a *k*-nearest-neighbor estimator (kNN),^37−42^ as previously described for rotational entropies.^28^

The mutual information expansion
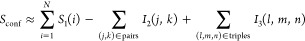
4is truncated after the three-molecule correlation term, where the indices 1 ≤ *i*, *j*, *k*, *l*, *m*, *n* ≤ *N* label individual molecules. The first term is the sum of all single-molecule entropies, i.e., the entropies of the marginal distributions of individual molecules, and therefore does not take correlations between molecules into account. Pairwise and triplewise correlations are described by the second and third terms, respectively, for which the mutual information terms read
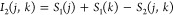
5a
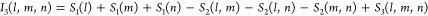
5bSimilar to the mutual information terms, also the entropy terms denote the entropies of the configuration space probability densities of one, two, and three water molecules, respectively. These are the entropies of the three-, six-, and nine-dimensional marginal distributions, respectively, where all degrees of freedom, except the ones of the molecules specified by the indices, are projected out. Because for a third-order expansion a nine-dimensional space needs to be sampled, the expansion converges for ∼10^5^ samples. Higher-order terms are increasingly harder to sample and were therefore neglected. While these may contribute to absolute entropies, we find for our examples that they tend to cancel out when calculating entropy differences.

The individual entropies of eqs 5a and 5b are calculated using a kNN estimator.^28^ The entropy of a given trajectory of *n*_*f*_ configurations {***x***_1_, ..., ***x***_*n*_*f*__} with , *p* = 1 (single-molecule term), *p* = 2 (pair term), or *p* = 3 (triple term) is given by
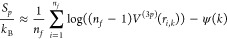
6where *k* is a fixed positive integer, ψ is the digamma function, *r*_*i*,*k*_ is the distance from the configuration ***x***_*i*_ to its *k*th neighbor in the 3*p*-dimensional configuration space using the Euclidean metric, and
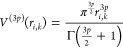
7is the volume of the (3*p* – 1)-sphere with radius *r*_*i*,*k*_.

The correlation term *I*_trans–rot_ is calculated as
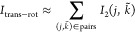
8where the index *j* denotes the translational degrees of freedom of molecule *j*, and *k̃* denotes the rotational degrees of freedom of molecule *k*.

To apply a kNN entropy estimator in the product space of  and the group of orientations SO(3), we use the composite metric
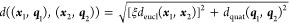
9where the quaternions ***q***_1_ and ***q***_2_ describe molecular orientations, *d*_eucl_ is the Euclidean metric, and *d*_quat_(***q***_1_, ***q***_2_) = min{∥***q***_1_ – ***q***_2_∥_2_, ∥***q***_1_ + ***q***_2_∥_2_} is the quaternion metric.^28,43^ The scaling factor ξ ensures equal units under the square root, where the distance in Euclidean space is measured in nanometers, and the distance in quaternion space is unitless. Its numerical value is chosen such that the typical distances in  and SO(3) are of the same magnitude, as discussed in greater detail in the Supporting Information. For liquid water, a value of ξ = 10 nm^–1^ is used, but tests with 20 and 30 nm^–1^ did not yield significantly different results.

The volume of the ball, induced by the metric *d* in  reads
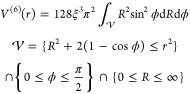
10 and was calculated numerically using the software Mathematica 10.0.^28,44^

## 3 Methods

MD simulations were carried out using the software package Gromacs 2018^45−49^ with a leapfrog integrator (2 fs time step) and the CHARMM36m force field.^50−52^ Bonds were constrained using SETTLE^53^ (water molecules) and LINCS^54^ (other bonds to hydrogen atoms). The V-rescale thermostat^55^ with a time constant of 0.1 ps at 300 K was used in all simulations, and NPT runs were performed using the Parrinello–Rahman barostat^56,57^ with a time constant of 1.0 ps and 1 bar of pressure. Lennard-Jones potentials^58^ were cut off at 1.2 nm. The same value was used as the real-space cutoff of electrostatic interactions in the particle-mesh Ewald (PME) method.^59^

All production trajectories used for the entropy estimates were 1 μs long; configurations were recorded every 10 ps.

### 3.1 Argon

To mimic the number density of liquid water, 1728 argon atoms were placed in a (4 nm)^3^ cubic box (equivalent to ∼10 000 bar pressure) and simulated under constant-temperature, constant-volume (NVT) conditions at a temperature of 300 K. Despite the large pressure, no crystallization occurred, and the system remained diffusive. Translational entropies were calculated using permutation reduction and a mutual information expansion (Per|Mut), and its accuracy was assessed using reference entropies obtained via the more expensive thermodynamic integration (TI).^13,14,28,31^

The TI was performed using 200 steps, during which the interactions were switched linearly from an ideal gas state (λ = 0), for which the entropy is known analytically, to full argon–argon interactions (λ = 1). The simulation runs for each step lasted 100 ns and were carried out with soft-core^60^ parameters α = 0.5 and *p* = 1. Errors were estimated as the difference to a second TI with only 50 ns per step.

Per|Mut entropies were calculated as described in Section 2. Permutation reduction of the 1 μs trajectory was carried out using a 12 × 12 × 12 simple cubic reference configuration {***r***_*i*_}. Pairwise MI terms were calculated for atoms with an average distance of less than 1.0 nm after permutation reduction. Similarly, a 0.45 nm cutoff was used for third-order MI terms. A kNN value of *k* = 1 was used for all MI terms. Error bars were estimated from the standard deviation of the entropies of the individual atoms.

**Figure 2:**
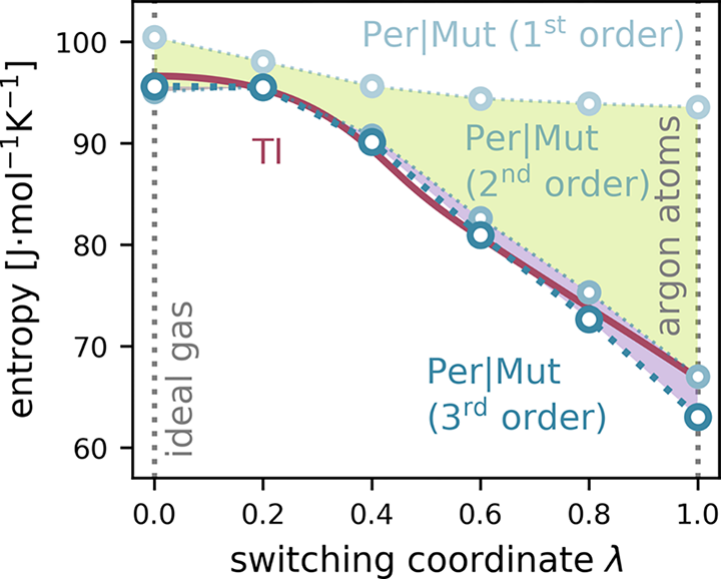
Entropy per particle of the argon system from TI (red) and Per|Mut (blue) along the switch from an ideal gas state (left) to full argon atoms (right). The MI expansions up to the first, second, and third order are shown with increasing opacity. The contribution by the second order, i.e., the difference between the first- and second-order expansion, is shaded in green. The contribution by the third order is shaded in magenta. Error bars are too small to be shown.

To compare the Per|Mut results for intermediate values of the TI switching coordinate, Per|Mut was also applied to 1 μs trajectories with λ-values of 0.8, 0.6, 0.4, 0.2, and 0.0 (ideal gas), as shown in Figure 2.

### 3.2 Alkanes and Octanol

Per|Mut entropies were calculated for the *n*-alkanes between ethane and decane as well as for octanol. Each solute molecule was solvated by 1728 water molecules in a cubic box and simulated as described above. To prevent the solutes from deviating from their initial linear configurations, the positions of all atoms were fixed, such that only the water molecules remained mobile. Permutation reduction was carried out using a 12 × 12 × 12 simple cubic reference configuration, and the MI terms were calculated using *k* = 1 as well as 1.0 and 0.33 nm cutoffs for second-order and third-order terms, respectively. Errors were estimated from the standard deviation of entropies of bulk-phase water molecules, assuming that molecules close to the solute are subject to the same spread.

For alkanes, reference entropies were again obtained using TI for ethane, propane, pentane, octane, and decane, where the solutes were grown in a water box during 200 steps for the Lennard-Jones interactions and an additional 50 steps for the Coulomb interactions. Each step lasted 50 ns, and soft-core parameters identical to that of the argon TI were used.

For the spatially resolved entropy map of octanol (Figure 4), the entropy per permutation-localized water molecule (see Figure 1C) was calculated by splitting the contributions from pair (second MI order) and triple correlations (third MI order) equally between the involved molecules. The simulation box was divided into 128 × 128 × 128 voxels, and for each voxel, the local entropy was given by the average of the entropies per water molecule, weighted according to the simulation trajectory.

### 3.3 Nearest-Neighbor Search

Nearest-neighbor searches for the kNN estimator in Euclidean space were performed using a k-d tree and the Python module scikit-learn 0.20.3.^61^ Nearest-neighbor searches in quaternion space and the composite space were carried out using the Non-Metric Space Library 1.7.3.6.^28,62^

## 4 Results and Discussion

### 4.1 Argon

To assess the accuracy of the translational entropies, we used a test system of 1728 argon atoms and compared entropies calculated with Per|Mut to entropies from thermodynamic integration (TI), as described in Section 3.1. TI is computationally more expensive than Per|Mut, does not yield spatial resolution, and is unsuitable for more complex systems but can serve as a reference for the test system. During TI, we changed the interactions between the argon atoms from a noninteracting ideal gas state (λ = 0) to their normal interatom interactions at λ = 1 and calculated the entropy change along the switching coordinate λ. We subsequently used Per|Mut to calculate translational entropies along the switching coordinate for λ = 0 (ideal gas), 0.2, 0.4, 0.6, 0.8, and 1.0 (full argon atoms) to compare with the respective reference values. As shown in Figure 2, Per|Mut closely follows the reference TI values for all λ-values with a maximum deviation of 5.7%.

For the ideal gas state, the third-order Per|Mut expansion yields a quite accurate entropy of 95.6 J·mol^–1^·K^–1^ and thus deviates by only 1% from the reference value 96.6 J·mol^–1^·K^–1^. In this state, the first order of Per|Mut contributes 100.4 J·mol^–1^·K^–1^, the second-order term reduces the entropy by 5.4 J·mol^–1^·K^–1^, and the third order contributes a further 0.5 J·mol^–1^·K^–1^.

As the atomic interactions are switched on, the reference TI entropy decreases by 29.8 to 66.7 J·mol^–1^·K^–1^ for λ = 1. The Per|Mut entropies follow the same trend; for full argon atoms, the third-order Per|Mut yields an entropy of 63.0 J·mol^–1^·K^–1^, which is within 5.7% from the reference value. The reduction of the first-order term (light blue in Figure 2) reflects the effect of the decreased accessible volume due to the excluded volumes of the interacting particles and amounts to just 6.9 J·mol^–1^·K^–1^, which is 21.1% of the total entropy loss. Significantly more entropy is lost due to correlated particle movement, as reflected by the second- and third-order contributions. Here, the pair correlations (second order) dominate by accounting for 21.2 J·mol^–1^·K^–1^, 65.0% of the overall entropy loss, whereas the three-particle correlations (third order) contribute 4.5 J·mol^–1^·K^–1^ (13.9%).

Since the particles in an ideal gas are by definition uncorrelated, it might seem surprising that for λ = 0, the second- and third-order contributions of Per|Mut are nonzero, due to the permutation reduction. As shown in Figure 1B, even an uncorrelated input distribution (left) may become correlated once the trajectory is mapped into a permutation subspace (right). The argon test system demonstrates that an MI expansion to the third order is sufficient to compensate the effect within 1% and that higher-order “pseudocorrelations” induced by permutation reduction are small. Furthermore, the second- and third-order “pseudocorrelations” for the ideal gas (5.4 and 0.5 J·mol^–1^·K^–1^, respectively) are small compared to the second- and third-order contributions for the full argon atoms (21.2 and 4.5 J·mol^–1^·K^–1^, respectively). Interpreting the contributions by the second and third order as measures of physical two- and three-body correlations is therefore still warranted for sufficiently interacting systems.

The significant but small third-order contribution of 4.5 J·mol^–1^·K^–1^ to the overall entropy loss shows that neglecting higher-order terms, which are expected to yield decreasing contributions, is justified.

As the interactions become stronger, the entropy is increasingly underestimated by up to 5.7%, likely because higher-order expansion terms become more important at high pressures.

Argon at ∼10 000 bar of pressure loses approximately 30 J·mol^–1^·K^–1^ of entropy compared to its ideal gas state, which is significantly more than the ∼18 J·mol^–1^·K^–1^ entropy loss of water compared to interactionsless water. Since kNN MI estimators are known to increasingly underestimate correlations the more correlated a system is,^28,63^ argon at high pressure poses a harder benchmark than water at 1 bar. We therefore expect the considered two- and three-body correlation terms to be more accurate for water than for argon.

Overall, Per|Mut yields accurate solvation entropies for the argon test system. To test the accuracy and the ability to provide spatially resolved entropies, we applied Per|Mut to more complex systems, which will be discussed in Sections 4.2 and 4.3.

### 4.2 Alkanes

Experimental and theoretical studies show that the solvation entropy of alkanes decreases approximately linearly with increasing alkane length.^64−66^ To see if Per|Mut captures this linear relationship qualitatively and quantitatively, we calculated hydration entropies for the *n*-alkanes from ethane to decane. Here, we defined the hydration shell as the 100 closest water molecules to the solute after permutation reduction. The number was chosen such that even for the largest solute (decane), all water molecules that were affected by its presence were still included (see Figure S3 in the Supporting Information). Reference values were obtained by TI, as described in Section 3.2.

**Figure 3:**
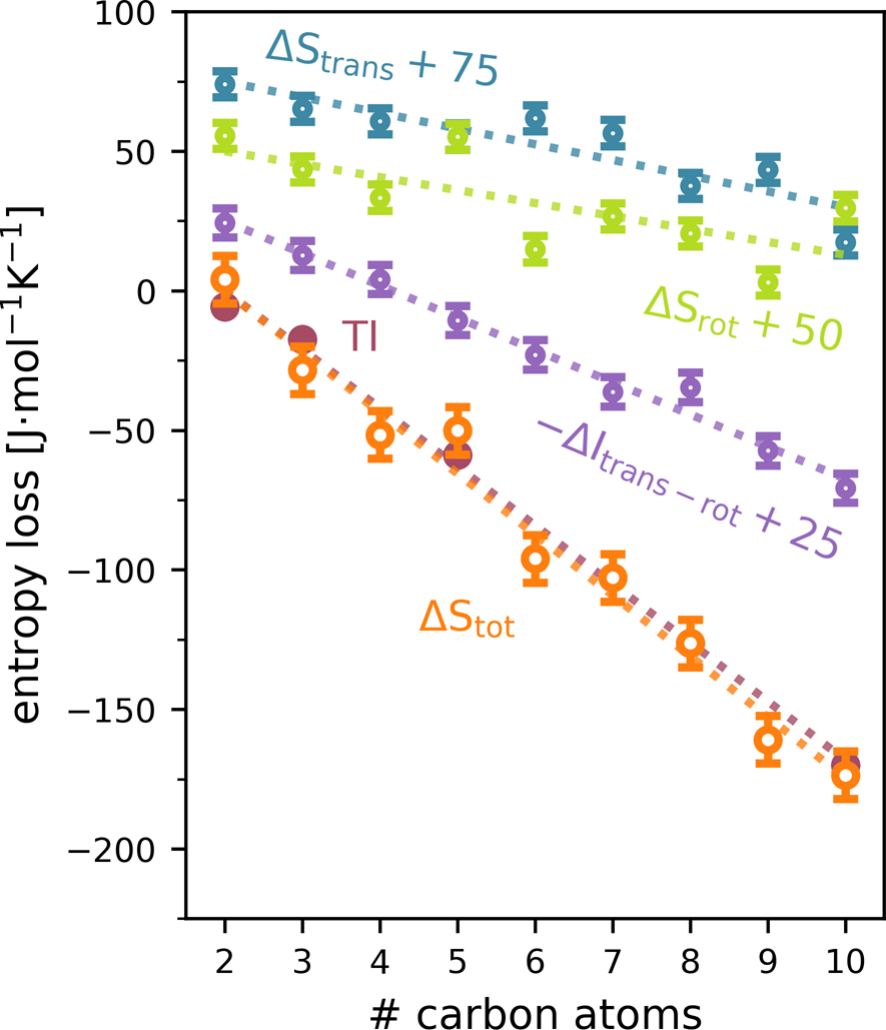
Hydration shell entropy loss relative to ethane with increasing alkane length. Translational and rotational entropies are illustrated with blue and green symbols, respectively. Entropy loss due to translation–rotation correlation is shown in purple. The total entropy change is shown in orange, and the TI reference entropies are shown in red. For easier visibility, the translational, rotational, and translation–rotation correlation data is offset by 75, 50, and 25 units, respectively. Dotted lines are from linear regression.

As shown in Figure 3, Per|Mut indeed yields linear trends for the translational (blue symbols) and rotational (green symbols) entropies as well as for the translation–rotation correlation term −Δ*I*_trans–rot_ (purple symbols), which reduce the entropy by (5.6 ± 1.0), (4.6 ± 1.7), and (11.6 ± 0.6) J·mol^–1^·K^–1^, respectively, for each additional carbon atom. Combined, this results in a loss of (21.8 ± 1.2) J·mol^–1^·K^–1^ per C atom (orange symbols). The result is in good agreement with the TI reference value of (21.0 ± 0.7) J·mol^–1^·K^–1^ per C atom (red symbols) and more than the experimental^64^ entropy loss of (13.3 ± 0.7) J·mol^–1^·K^–1^.

Since the difference between TI and experimental values is most likely due to force field errors, which equally affect Per|Mut, we consider TI to be the proper benchmark.

Notably, increased correlations between translational and rotational water motions for longer alkanes reduce the entropy by as much as the combination of translational and rotational modes. The correlation of translational and rotational modes of motion increases for molecules close to the solute. In this regime, the molecules likely experience an increased orientational bias by predominately forming hydrogen bonds facing away from the solute. By this reasoning, larger solutes result in a larger entropy loss from the correlation term.

Overall, Per|Mut accurately calculates the solvation entropy change for alkanes between ethane and decane. Furthermore, the method precisely captures the entropy change, induced by the addition of a chemical group as small as a methyl group to the solute for a hydration shell of 100 molecules.

### 4.3 Octanol

Our approach allows closer analysis of the molecular origin of entropy changes. To assess the spatial resolution yielded by Per|Mut, we calculated the hydration shell entropy of octanol. To this end, we simulated a fixed octanol molecule with 1728 water molecules in a similar manner as described in Section 3, carried out the Per|Mut analysis, and calculated local entropies as described in Section 3.2.

**Figure 4:**
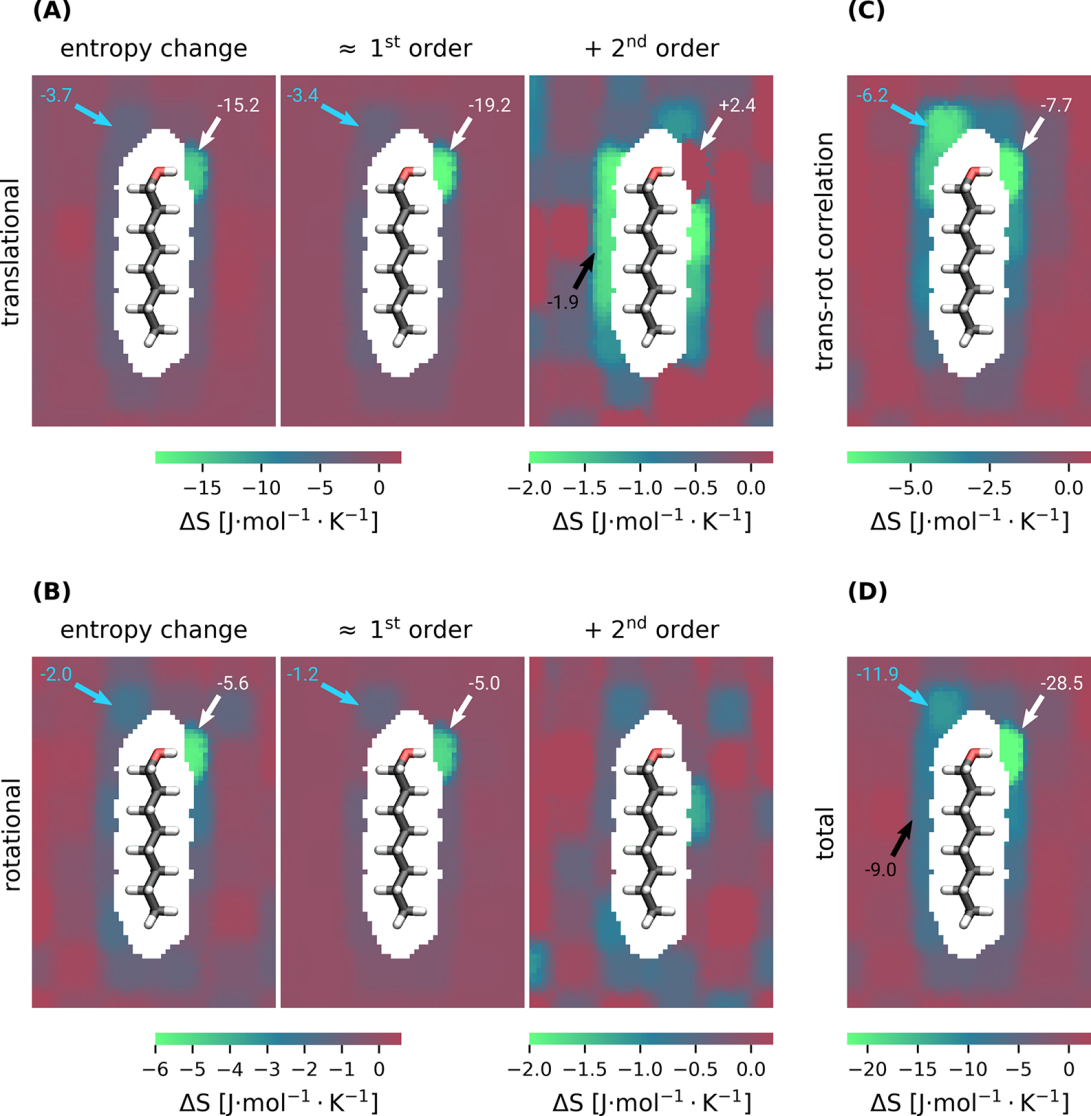
Spatially resolved octanol hydration shell entropy change per molecule relative to bulk quantities. (A) shows translational entropy values, and (B) shows rotational entropies. In both, (A) and (B), the three columns show total entropy change, first-order MI change, and second-order MI change, respectively. (C) shows the translation–rotation correlation, and (D) shows the total entropy change. Values at selected regions are highlighted with arrows.

As shown in Figure 4, the spatial distribution of entropy differs significantly between the apolar tail and in the vicinity of the OH group of octanol.

The translational entropy (Figure 4A) is reduced by 15.2 J·mol^–1^·K^–1^ per water molecule where the hydroxyl group acts as a proton donor (white arrow) and by 3.7 J·mol^–1^·K^–1^ where it acts as a proton acceptor (cyan arrow). Close to the hydrophobic tail, the entropy reduction varies between 3.7 and 1.5 J·mol^–1^·K^–1^.

The MI expansion allows for the entropy decomposition into contributions from the individual water molecules (first order) and their entropy loss due to pair correlations (second order). The first-order contribution (Figure 4A, center) shows that an entropy loss of 19.2 J·mol^–1^·K^–1^ at the donor side of the hydroxyl group is due to the reduced mobility of a hydrogen-bonded water molecule. At the acceptor side, only 3.4 J·mol^–1^·K^–1^ is lost. The entropy loss around the tail is mainly from the first-order contribution, where the presence of the apolar hydrocarbon chain causes an entropy loss up to 3.5 J·mol^–1^·K^–1^ due to the restricted mobility of individual water molecules.

As seen for the second-order contribution (Figure 4A, right), the entropy loss due to pair correlations of the water molecule that forms a hydrogen bond to the hydroxyl group is less than in bulk phase. The molecule therefore gains 2.4 J·mol^–1^·K^–1^ relative to bulk (white arrow). A likely explanation is that the hydroxyl group replaces another water molecule as a hydrogen bond partner, which leaves the chemical environment almost unchanged but reduces the number of possible water–water correlation pairs in the vicinity. Furthermore, increased pairwise correlations decrease the entropy in a shell around the hydrophobic chain by additional 1.9 J·mol^–1^·K^–1^ per molecule (black arrow). This result is unexpected, as water molecules close to the octanol molecule have fewer neighbors, thus showing that correlations with the remaining neighbors disproportionally increase at the hydrophobic tail.

The third-order correlation does not show significant spatial heterogeneity and, therefore, is not included in Figure 4.

The rotational entropy (Figure 4B) behaves similarly, albeit its contributions are smaller. 5.6 J·mol^–1^·K^–1^ is lost by the water molecule for which the OH group of octanol acts as the proton donor (white arrow), to which the first order contributes 5.0 J·mol^–1^·K^–1^. On the acceptor side, 2.0 J·mol^–1^·K^–1^ is lost, to which the first order contributes 1.2 J·mol^–1^·K^–1^ (cyan arrows). Near the hydrocarbon chain, the rotational entropy is reduced by ∼1.6 J·mol^–1^·K^–1^ per molecule, of which ∼1.0 J·mol^–1^·K^–1^ is due to a hindered rotational motion of the individual water molecules (first order). Again, there is a shell of more correlated water molecules around the hydrophobic part of octanol (second order), which is less pronounced than that for the translational entropy.

The mutual information on translational and rotational degrees of freedom (Figure 4C) shows strong correlations, equivalent to an entropy loss of 7.7 J·mol^–1^·K^–1^ at the donor site (white arrow) and 6.2 J·mol^–1^·K^–1^ at the acceptor site (cyan arrow). Furthermore, the translation–rotation correlations reduce the entropy of each molecule close to the apolar chain by 1.0 to 3.5 J·mol^–1^·K^–1^.

As shown in Figure 4D, the total hydration entropy of octanol is mainly affected by the polar hydroxyl group, where entropy is reduced by 28.5 J·mol^–1^·K^–1^ (white arrow) and 11.9 J·mol^–1^·K^–1^ (cyan arrow) at the donor and acceptor sites, respectively.

The discrepancy between the two hydrogen binding sites is most likely caused by different bond strengths. Whereas hydrogen atoms of the hydroxyl group and of a water molecule carry almost identical partial charges, the oxygen atom of the hydroxyl group carries a partial charge of −0.65 elementary charges, significantly less than a water oxygen atom (−0.834 elementary charges).

In addition, the entropy of each water molecule close to the hydrocarbon chain is reduced by 9 J·mol^–1^·K^–1^, which results from both a loss of mobility of individual molecules (first order) and increased correlations at the surface of the solute.

A quantitative comparison between the hydrophilic hydroxyl group and the apolar tail (or, equivalently, between octanol and octane) yields a solvent entropy difference of (36 ± 3) J·mol^–1^·K^–1^, which was determined using eight-molecule shells around the OH group and its hydrophobic counterpart, following the same rationale as for the alkanes. The result is significantly larger but comparable in magnitude to the TI reference estimate of (25.1 ± 0.1) J·mol^–1^·K^–1^ (see the Supporting Information). Aside from possible sampling issues, the deviation is likely the result of omitted higher-order correlations, which are affected differently by the polar hydroxyl group and the apolar chain.

## 5 Conclusions

We developed Per|Mut, a new method to calculate hydration entropies of water, and assessed its accuracy on argon, alkanes, and octanol test systems.

Our method rests on a permutation reduction^31,32^ (Section 2.2), which alleviates the sampling problem by *N*! and localizes the water molecules (Figure 1C), leaving the physics of the system unchanged. Due to the localization of the molecules, spatially resolved entropies can be calculated at the level of single water molecules. Further, a MIE is employed, which allows the absolute entropy to be decomposed into contributions from individual molecules, pair correlations, and triple correlations. The MIE reduces the dimensionality of the spaces that need to be sampled. By distinguishing between entropy contributions of individual molecules as well as pairwise and triple correlations, additional insight into the physical origin of entropy changes is provided.

We used the small argon test system to assess the accuracy of the translational entropy algorithm by comparing the obtained values with TI. Per|Mut yielded accurate entropy values for the full range of the switching coordinate within a maximum deviation of 5.7% from the TI reference value.

To test the accuracy of Per|Mut as a whole, including the translation–rotation correlation term, we calculated the hydration entropies of *n*-alkanes from ethane to decane.

Indeed, we identified a linear entropy loss^66^ of (21.8 ± 1.2) J·mol^–1^·K^–1^ per additional carbon atom, as shown in Figure 3, which is in quantitative agreement with the reference entropy loss of (21.0 ± 0.7) J·mol^–1^·K^–1^ per C atom, calculated by TI. Here, the increased correlation between the translational and rotational degrees of freedom for larger solutes was identified as the largest contribution to the entropy loss.

Because of its hydrophobic tail and its hydrophilic headgroup, we chose octanol as a test system to demonstrate how Per|Mut can characterize solvation entropies with a spatial resolution. Hydrogen bonding strongly reduces the local entropy by 11.9 and 28.5 J·mol^–1^·K^–1^ for the water molecules to which the hydroxyl group of octanol acts as a proton acceptor or donor, respectively. The entropy loss at the donor site yields an entropic free energy contribution of 8.55 kJ·mol^–1^ at a temperature of 300 K, which is significantly less than the ∼20 kJ·mol^–1^ enthalpy loss due to the hydrogen bond.^67^ The result shows that the solvation free energy difference of octanol and octane is enthalpy-driven.

Near the hydrophobic tail of octanol, the entropy is reduced for both the first- and second-order MI term. The losses of up to 1.9 J·mol^–1^·K^–1^ due to translational correlations and up to 3.5 J·mol^–1^·K^–1^ due to correlations between translation and rotation show that the lack of strong interactions with the apolar octanol tail causes stronger interactions within the remaining water, an effect that is similarly discussed in previous publications.^68,69^ The finding does not necessarily imply an increased structural order in the hydration shell, as predicted by the controversial iceberg model,^70^ but identifies a reduced single-molecule mobility (first order) and increased water correlations (second order) as main causes for the entropy loss.

For both the alkane and octanol systems, the solutes were immobilized to obtain an unblurred spatial resolution and to eliminate solute–solvent correlations, which are not captured by the method but would contribute to the solvent-related entropy. In scenarios where flexible-solute effects need to be included, an ensemble-averaged entropy can be obtained by carrying out multiple entropy calculations for a representative sample of the ensemble of solute configurations (e.g., taken from a seeding trajectory).

As seen in Section 4.3, Per|Mut overestimates the entropy loss of octanol compared to octane. Likewise, Per|Mut yields absolute water entropies of around 106 J·mol^–1^·K^–1^ per molecule, which is higher than the known absolute water entropy^71^ of ∼70 J·mol^–1^·K^–1^. Since the TIP3P water model has proven to reproduce this value,^16^ we tentatively attribute this deviation to higher-order correlations, which we neglected. This is not a fundamental limitation of the method; however, higher than 3-body correlation terms are hard to converge with realistic sample sizes. As our results for octanol indicate, entropy differences appear to be more accurate. For hydrophobic surfaces like alkanes, accurate entropy differences were obtained.

Importantly, due to the decomposition into one-, two-, and three-body correlation terms, Per|Mut provides an intuitive and spatially resolved picture of entropy changes, which allows the solvent effects of individual chemical groups or protein side chains to be identified and assessed. Due to the locality of the water molecules after permutation reduction, entropies of small subsystems, like the solvation shells of alkanes or—potentially—a ligand binding site, can be calculated without having to consider the entire system, which reduces statistical errors.

Our implementation of Per|Mut is available for download as a Python package (https://gitlab.gwdg.de/lheinz/hydration_entropy).

## Supporting Information Available

Detailed mathematical derivation of the entropy separation into translational, rotational, and translation–rotation correlations. Additional explanations and simulation results (PDF)
