# Supplementary material for: Per|Mut: Spatially Resolved Hydration Entropies from Atomistic Simulations
Source: J Chem Theory Comput. 2021 Mar 12;17(4):2090–8. doi: 10.1021/acs.jctc.0c00961 (PMC8047778; doi:10.1021/acs.jctc.0c00961)
Supplement: Supplementary file 1 — ct0c00961_si_001.pdf [file ct0c00961_si_001.pdf]

Supplementary material:

Per|Mut: Spatially resolved hydration entropies from atomistic simulations

Leonard P. Heinz* and Helmut Grubmüller*

Department of Theoretical and Computational Biophysics, Max-Planck Institute for Biophysical Chemistry, Göttingen, Germany

E-mail: lheinz@gwdg.de; hgrubmu@gwdg.de

The mutual information expansion of hydration entropy

We describe the configuration of N water molecules using N translational degrees of freedom from $\mathbb{R}^3$, and N orientations from $SO(3)$. The set of translational degrees of freedom is $\mathcal{T} = \{1, \ldots, i, \ldots, N\}$, where the index i denotes the translational coordinates of molecule i. In the same fashion, the set of rotational degrees of freedom is $\mathcal{R} = \{1, \ldots, j, \ldots, N\}$.

The 3rd-order mutual information expansion of the total system entropy is defined as[1–4]

$$S_{\text{tot}} \approx \sum_{i\in(\mathcal{T}\cup\mathcal{R})} I_1(i) - \sum_{(j,k)\in\text{Pairs}(\mathcal{T}\cup\mathcal{R})} I_2(j,k) + \sum_{(l,m,n)\in\text{Triples}(\mathcal{T}\cup\mathcal{R})} I_3(l,m,n), \qquad (1)$$

where $(j, k) \in \text{Pairs}(\mathcal{T} \cup \mathcal{R})$ is a pair with $i \neq j$, and $(l, m, n) \in \text{Triples}(\mathcal{T} \cup \mathcal{R})$ is a triple of degrees of freedom with unique l, m, n.

The entropy from equation 1 is split into rotational, translational, and mixed parts:

$$S_{\text{tot}} \approx \sum_{i\in\mathcal{T}} I_1(i) + \sum_{\tilde{i}\in\mathcal{R}} I_1(\tilde{i}) \tag{2}$$

$$- \sum_{(j,k)\in\text{Pairs}(\mathcal{T})} I_2(j,k) - \sum_{(\tilde{j},\tilde{k})\in\text{Pairs}(\mathcal{R})} I_2(\tilde{j},\tilde{k}) - \sum_{j\in\mathcal{T},\ \tilde{k}\in\mathcal{R}} I_2(j,\tilde{k}) \tag{3}$$

$$+ \sum_{(l,m,n)\in\text{Triples}(\mathcal{T})} I_3(l,m,n) + \sum_{(\tilde{l},\tilde{m},\tilde{n})\in\text{Triples}(\mathcal{R})} I_3(\tilde{l},\tilde{m},\tilde{n}) \tag{4}$$

$$+ \sum_{l\in\mathcal{T},\ (\tilde{m},\tilde{n})\in\text{Pairs}(\mathcal{R})} I_3(l,\tilde{m},\tilde{n}) + \sum_{(l,m)\in\text{Pairs}(\mathcal{T}),\ \tilde{n}\in\mathcal{R}} I_3(l,m,\tilde{n}). \tag{5}$$

The translational entropy reads

$$S_{\text{trans}} \approx \sum_{i\in\mathcal{T}} I_1(i) - \sum_{(j,k)\in\text{Pairs}(\mathcal{T})} I_2(j,k) + \sum_{(l,m,n)\in\text{Triples}(\mathcal{T})} I_3(l,m,n), \tag{6}$$

and the rotational entropy reads

$$S_{\text{rot}} \approx \sum_{\tilde{i}\in\mathcal{R}} I_1(\tilde{i}) - \sum_{(\tilde{j},\tilde{k})\in\text{Pairs}(\mathcal{R})} I_2(\tilde{j},\tilde{k}) + \sum_{(\tilde{l},\tilde{m},\tilde{n})\in\text{Triples}(\mathcal{R})} I_3(\tilde{l},\tilde{m},\tilde{n}). \tag{7}$$

The translation-rotation correlation contribution is given by the remaining mixed terms and reads

$$I_{\text{trans-rot}} \approx \sum_{j\in\mathcal{T},\ \tilde{k}\in\mathcal{R}} I_2(j,\tilde{k}) - \sum_{l\in\mathcal{T},\ (\tilde{m},\tilde{n})\in\text{Pairs}(\mathcal{R})} I_3(l,\tilde{m},\tilde{n}) - \sum_{(l,m)\in\text{Pairs}(\mathcal{T}),\ \tilde{n}\in\mathcal{R}} I_3(l,m,\tilde{n}). \tag{8}$$

In this Per|Mut implementation, we neglect the mixed three-body correlation terms due to their small contribution and slow convergence.

Using this separation, the full entropy is

$$S_{\text{tot}} = S_{\text{trans}} + S_{\text{rot}} - I_{\text{trans-rot}}. \tag{9}$$

Choosing the scaling factor ξ for the composite metric

The composite metric

$$d((\boldsymbol{x}_1, \boldsymbol{q}_1), (\boldsymbol{x}_2, \boldsymbol{q}_2)) = \sqrt{\left[\xi d_{\text{eucl}}(\boldsymbol{x}_1, \boldsymbol{x}_2)\right]^2 + d_{\text{quat}}(\boldsymbol{q}_1, \boldsymbol{q}_2)^2}, \tag{10}$$

defined in eq. 8 of the main text, measures combined distances between molecular positions in Euclidean space $\boldsymbol{x}$ and molecular orientations $\boldsymbol{q}$. The scaling factor ξ serves to ensure equal units under the square root, where d_{eucl} yields distances in nm and d_{quat} returns unitless orientational distances. In the limit of infinite sampling, its numerical value is irrelevant, however, practical considerations limit it to a reasonable range at finite sampling.

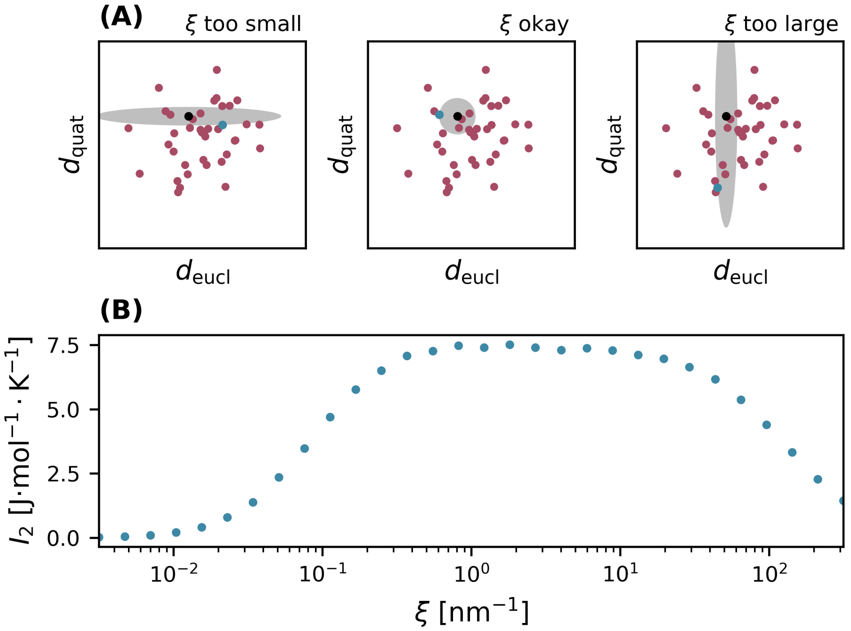

Figure S1: (A) Qualitative illustrations of the effects of too small ξ, reasonably chosen ξ, and too large ξ (from left to right) using an example data set. The horizontal and vertical axes symbolize the translational degrees of freedom (in Euclidean space) and the orientational degrees of freedom (using quaternions), respectively. The grey ellipses visualize the balls induced by the respective (ξ-dependent) metric around the black data point to its $k = 5$-nearest neighbor (blue). (B) The mutual information between the translational and rotational degrees of freedom of a water molecule close the the hydroxyl group of octanol in dependence of the scaling factor ξ. The correct value of $\approx 7.4\,\text{J}\cdot\text{mol}^{-1}\cdot\text{K}^{-1}$ is reached in a plateau region between $\xi \approx 0.2$ and 30 using $k = 1$.

As shown in Fig. S1, too small or too large scaling factors ξ result in strongly elongated k-nearest neighbor balls, either along the translational or the orientational degrees of freedom. Since the k-nearest neighbor method estimates the local probability density as k over the volume of the ball to the kth neighbor, the probability density is assumed to be constant within each ball. For too elongated balls, this is no longer the case (see Fig. S1A). As a result, mutual information is underestimated, as correlations are "smeared out".

This effect is demonstrated in Fig. S1B, where the mutual information between translational and rotational degrees of freedom of a water molecule close the the hydroxyl group of octanol were calculated for scaling factors ξ between $10^{-2.5}$ and $10^{+2.5}\,\mathrm{nm}^{-1}$. As expected, the mutual information is underestimated for very small and very large values, whereas the correct value of $\approx 7.4\,\mathrm{J{\cdot}mol^{-1}{\cdot}K^{-1}}$ is reached in a plateau region between $\xi \approx 0.2$ and 30. As the width of the distribution of a single water molecule after permutation reduction is $\approx 0.3\,\mathrm{nm}$ and the maximal orientational distance d_{quat} is $\sqrt{2} \approx 1.41$,[5,6] the plateau region corresponds to the order of magnitude where the balls approximately reflect the elongation of the expected data set.

Here, a fixed scaling factor of $\xi = 10\,\mathrm{nm}^{-1}$ was chosen within the plateau region of suitable values.

Liquid argon at 120 K and 1 bar

To mimic the number density of water, the argon test system was simulated at an average pressure of 10000 bar. Although the system behaves as a liquid in the simulation, these conditions represent a metastable region in the phase diagram. We therefore carried out an additional analysis at liquid-argon conditions of 120 K and a pressure of 1 bar. Because under these conditions, the number density is slightly smaller than that of water, the pair correlation and triple correlation cut-offs were increased to 1.15 nm and 0.5 nm, respectively.

As shown in Fig. S2, the entropy yielded by Per|Mut is accurate within 1.3 % of the TI

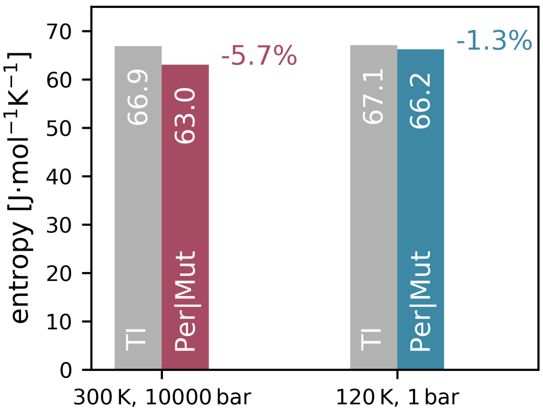

Figure S2: Comparison between the TI reference values (grey) and the third-order Per|Mut entropies of the argon test system at high pressure (red) and the same system at a temperature of 120 K and a pressure of 1 bar (blue).

reference value.

Choosing the hydration shell around alkanes

To compare the entropy losses associated with the solvation of various alkanes, comparison needs to be made considering the same number of water molecules around each alkane. We therefore defined a solvation shell, containing the closest n water molecules (after permutation reduction) around each solute. The solvation shells need to be large enough that all water molecules that are affected by the solutes are included in the calculations, even for the largest solute (decane). To determine the optimal solvation shell size n, we calculated the alkane entropy differences for the closest 20, 50, 100, 200, 300, and 500 water molecules. As shown in Fig. S3A, the slope of the entropy loss (average entropy loss per additional C-atom) converges once the closest 100 water molecules are considered, indicating that all relevant solvation shell entropy effects are included. For larger solvation shells (e.g., the closest 200, 300, or 500 molecules), the average entropy loss per additional C-atom remains almost unchanged, but the statistical error increases, as more bulk water molecules are (unnecessarily) included in the calculation. We therefore considered the closest 100 water molecules the

appropriate hydration shell.

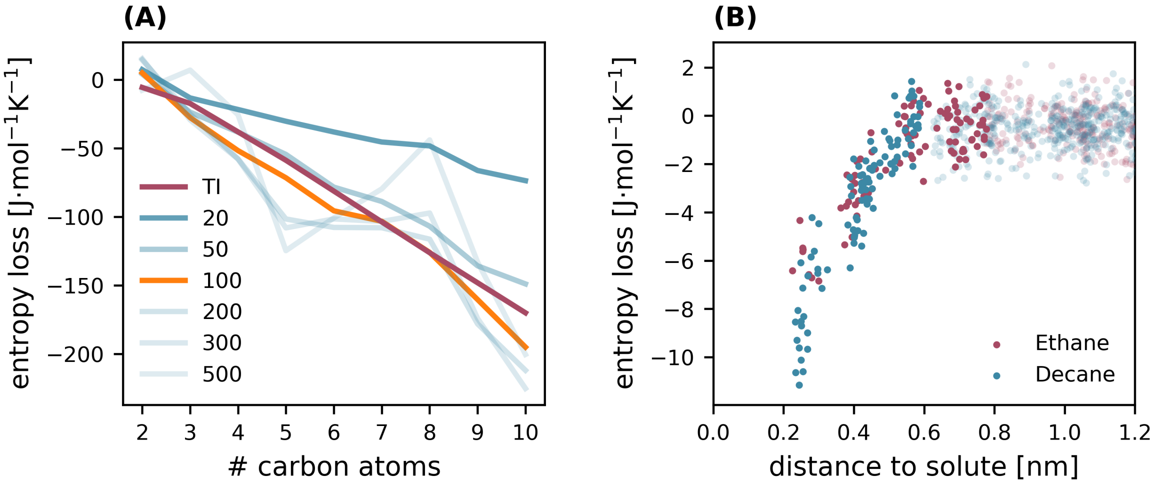

Figure S3: (A) Total hydration shell entropy loss relative to ethane with increasing alkane length, as shown in Fig. 3 of the main text. TI reference values are shown in red. Per|Mut entropy losses of the closest 20 to 500 water molecules are shown in blue and orange. (B) The entropy loss per water molecule (relative to bulk) in dependence on the distance to the solute for both, Ethane (smallest solute) and Decane (largest solute). The entropies of the 100 closest water molecules are shown in full color, further water molecules are shown as semi-transparent.

Indeed, as shown in Fig. S3B, the 100-molecule shell just encapsulates all water molecules that are affected by the largest solute (Decane). This shell size therefore captures all solvation-effects, but minimizes the statistical error by excluding unnecessary bulk water molecules.

Octanol TI reference entropy

Initial attempts to determine the solvation entropy difference between octane and octanol through thermodynamic integration (TI) have proven difficult, as the entropy change due to the Coulomb-interactions in particular could not be sufficiently converged. Contrary to the widely used free-energy formulation of TI, entropy TI generally suffers from poor convergence properties.

To overcome this problem, we instead calculated the solvation entropy difference between

propane and propanol, assuming that the entropy change due to the addition of the hydroxyl group is essentially independent of the hydrocarbon chain length. This allowed us to reduce the system size from initially 1728 water molecules to only 547 molecules, while a distance of at least 1 nm was kept between the centered solute and the boundaries of the simulation box. 250 intermediate λ-windows were simulated, of which each lasted 1 μs. We obtained an entropy difference of $(25.1 \pm 0.1)\,\mathrm{J{\cdot}mol^{-1}{\cdot}K^{-1}}$, where the error was determined by omitting 500 ns from each window.

The result serves as an estimate for the entropy difference between octane and octanol.

References

(1) Matsuda, H. Physical nature of higher-order mutual information: Intrinsic correlations and frustration. *Physical Review E* **2000**, *62*, 3096.

(2) Hnizdo, V.; Darian, E.; Fedorowicz, A.; Demchuk, E.; Li, S.; Singh, H. Nearest-neighbor nonparametric method for estimating the configurational entropy of complex molecules. *Journal of computational chemistry* **2007**, *28*, 655–668.

(3) Hnizdo, V.; Tan, J.; Killian, B. J.; Gilson, M. K. Efficient calculation of configurational entropy from molecular simulations by combining the mutual-information expansion and nearest-neighbor methods. *Journal of computational chemistry* **2008**, *29*, 1605–1614.

(4) Fengler, M. Estimating Orientational Water Entropy at Protein Interfaces. Ph.D. thesis, Georg-August-Universität Göttingen, 2011.

(5) Heinz, L.; Grubmüller, H. Computing spatially resolved rotational hydration entropies from atomistic simulations. *Journal of Chemical Theory and Computation* **2019**, *16*, 108–118.

(6) Huynh, D. Q. Metrics for 3D rotations: Comparison and analysis. *Journal of Mathematical Imaging and Vision* **2009**, *35*, 155–164.
